# Supplementary material for: C. elegans Runx/CBFβ suppresses POP-1 TCF to convert asymmetric to proliferative division of stem cell-like seam cells
Source: Development. 2019 Nov 18;146(22):dev180034. doi: 10.1242/dev.180034 (PMC6899014; doi:10.1242/dev.180034)
Supplement: Supplementary information [file develop-146-180034-s1.pdf]

SUPPLEMENTARY INFORMATION

FIGURE S1

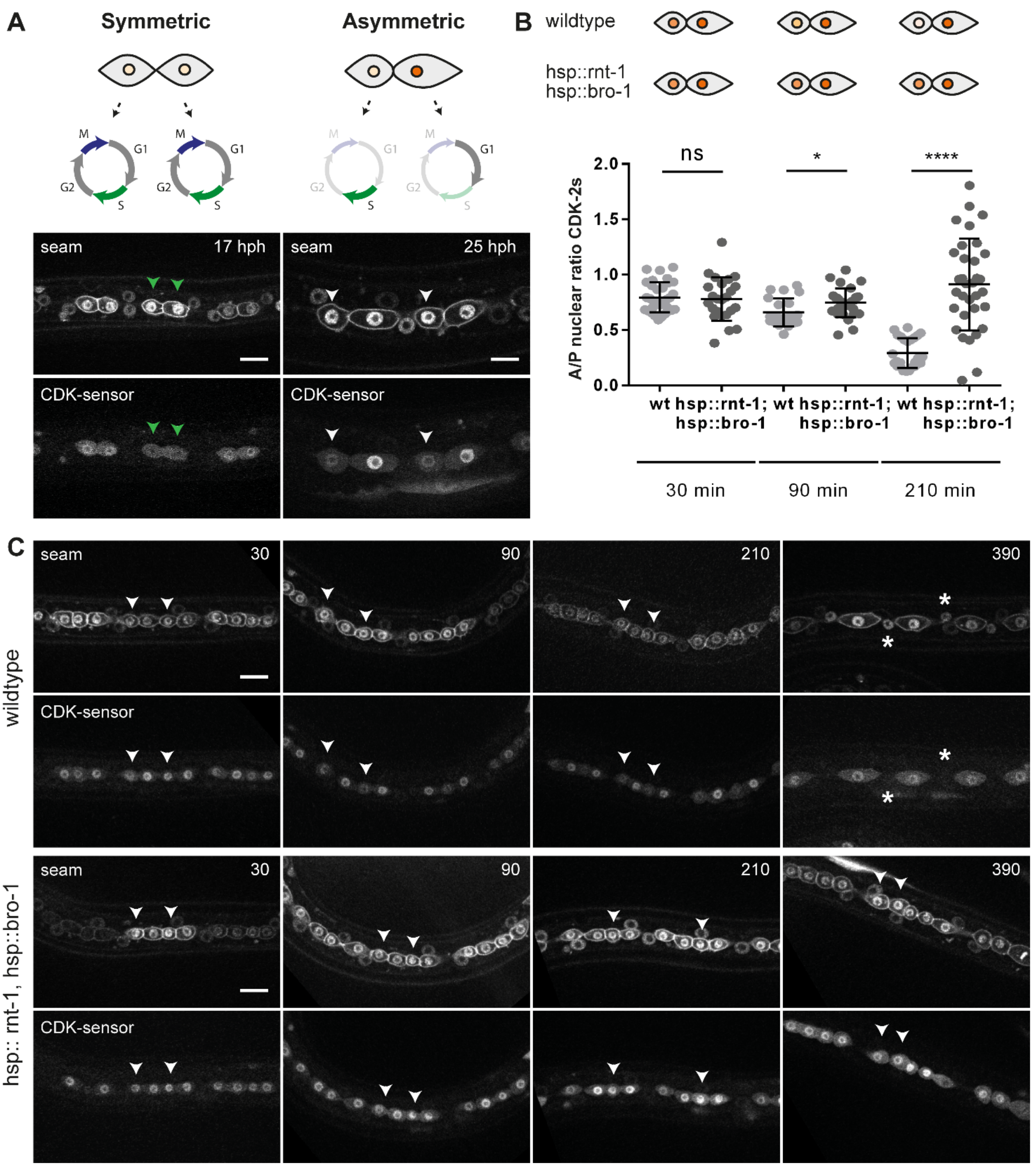

Figure S1. Cell cycle progression as a read-out for daughter cell fate. (A) Seam cells progress differently through the cell cycle after symmetric or asymmetric cell division (Top), as illustrated by spinning disk microscopy images of seam cells expressing the CDK sensor (Bottom). Left panels: seam daughter cells after symmetric L2 division, t=17 hours post hatching (hph). Green arrowheads point to the daughter cells of the V2.p symmetric division.

Development • Supplementary information

Right panels: daughter cells after asymmetric division in L3, t=25 hph. White arrowheads indicate anterior daughter cells of the V3.pap and V3.ppp divisions. Note that the CDK sensor helps to distinguish between anterior fate (cell cycle reentry; nuclear export of GFP) and seam cell self-renewal (quiescence; nuclear retention of GFP). (B) A/P nuclear ratio of the CDK-2 sensor in control and heat-shock induced RNT-1/BRO-1 L2 seam cells measured at different timepoints after induction. Time indicates minutes after heat shock, which was stopped just prior to or during mitosis. (C) Spinning disk microscopy of control and heat-shock induced RNT-1/BRO-1 seam cells. Images taken at different time point after induction. White arrowheads point to anterior daughter cells of L2 asymmetric Vn.pa and Vn.pp divisions. Asterisks indicate anterior daughter cells that have fused with the hypodermis. Images were processed using ImageJ software. Scale bars represent 10 µm. Error-bars represent mean ± SD.

Figure S2

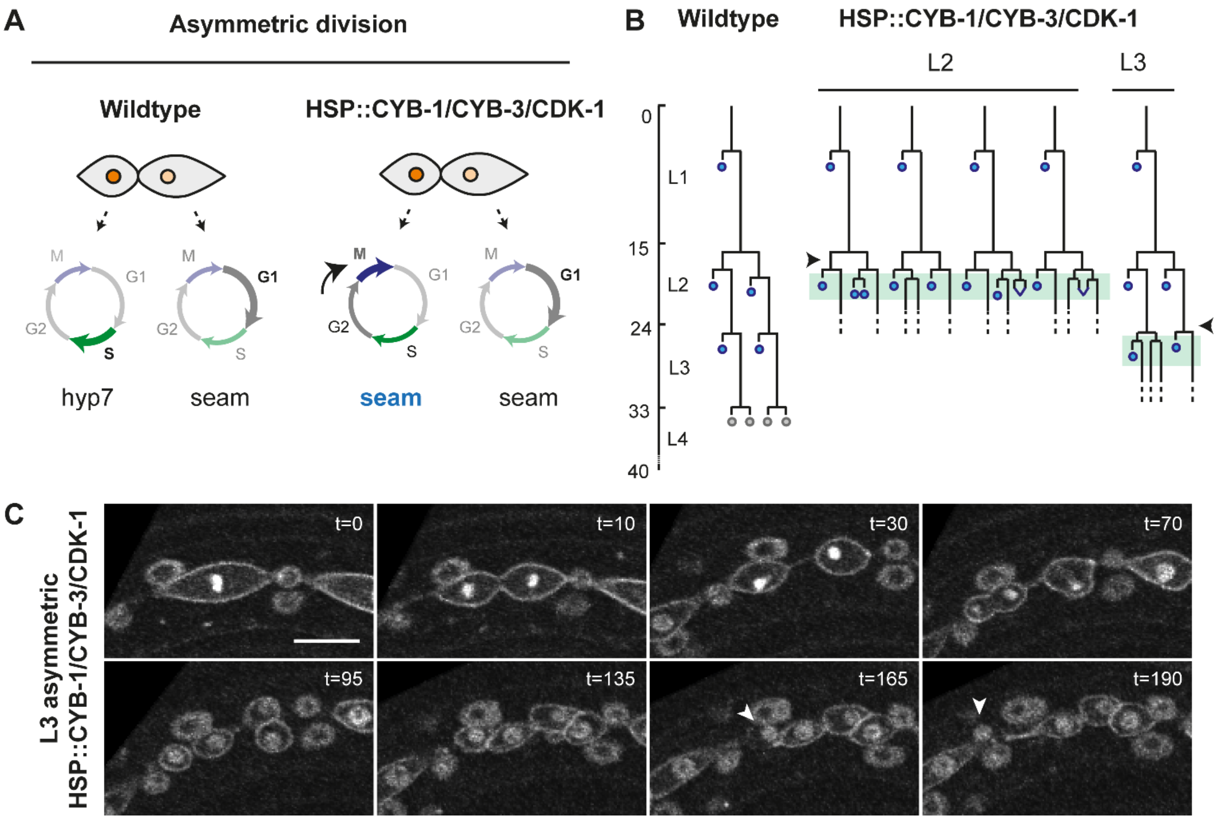

Figure S2. Induced expression of CDK-1, CYB-1, and CYB-3 occasionally induced extra mitosis. (A) Seam daughters of a wild-type asymmetric division progress differently after cytokinesis: anterior differentiating daughter cells undergo S-phase before fusing with the hyp7 epidermis. Posterior daughters pause in G1 until the next larval stage. Heat-shock induced expression of *cdk-1, cyb-1,* and *cyb-3* was used to force anterior daughter cells of asymmetric divisions to progress into mitosis after completing S-phase. We hypothesized that cell cycle progression of the anterior cells might overrule differentiation into hyp7 and trigger these cells to maintain a seam fate (blue). (B). Lineage analyses of L2 (middle) and L3 (right) animals with heat-shock induced CDK-1/CYB-1/CYB-3. Heat shock was given between the symmetric and asymmetric L2 division (middle; arrowhead) or prior to the L3 asymmetric division (right; arrowhead). Animals were followed using time-lapse microscopy during the hours after heat shock (green boxes). (C) Time-lapse spinning disk microscopy of L3 animals with heat-shock induced CDK-1/CYB-1/CYB-3. Time-lapse represents the L3 lineage presented in panel B. Both the anterior and posterior daughter cells of the L3 asymmetric division undergo an additional mitosis, of which the anterior daughter cell of the anterior division differentiates and fuses with the epidermis (arrowhead). Images were processed using ImageJ software. Scale bar represents 10 µm.

Development • Supplementary information

FIGURE S3

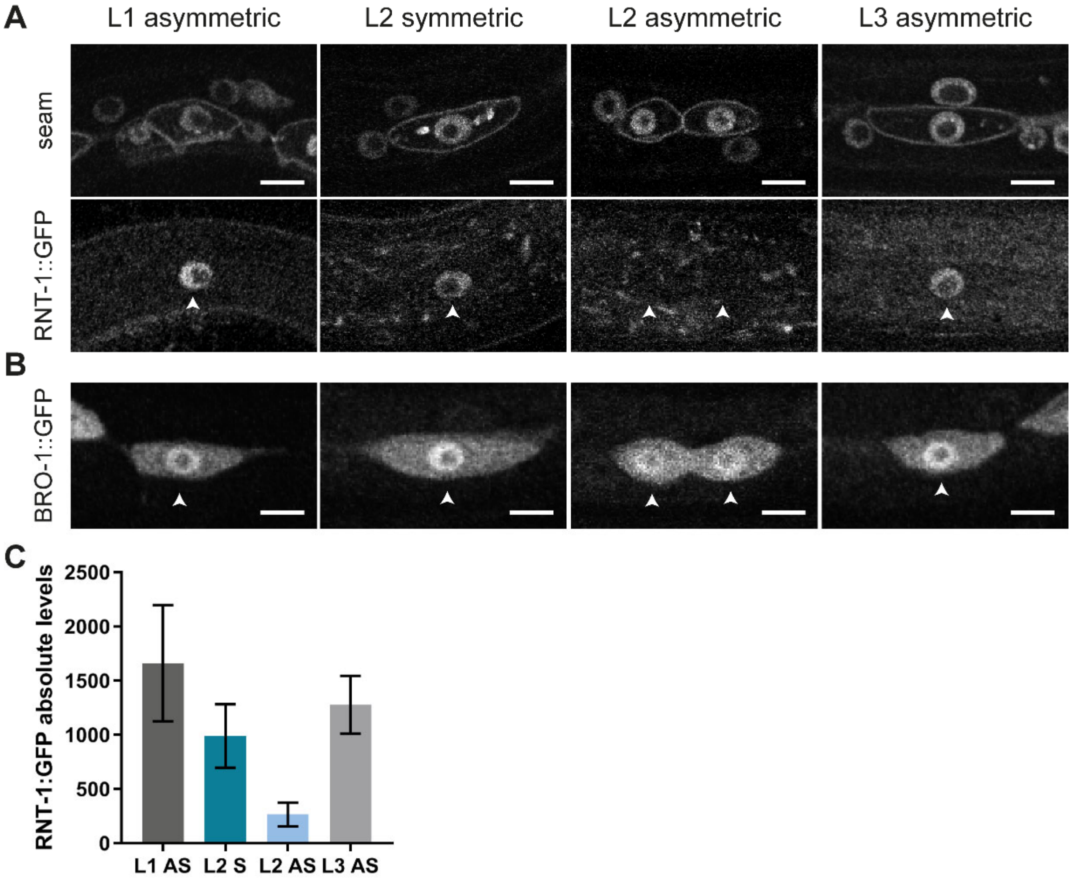

Figure S3. Endogenous RNT-1 and transgenic BRO-1 expression during seam cell development. (A) Spinning disk images of endogenous RNT-1::GFP expression prior to nuclear envelope breakdown in L1-L3 seam cells. Seam markers are mCherry::PH and mCherry::H2B. Arrowheads point to nuclear RNT-1::GFP. (B) Spinning disk images of transgenic BRO-1::GFP prior to nuclear envelope breakdown in L1-L3 seam cells. Arrowheads point to nuclear BRO-1::GFP. (C) Quantification of the levels of endogenous RNT-1::GFP in L1-L3 seam cells.

FIGURE S4

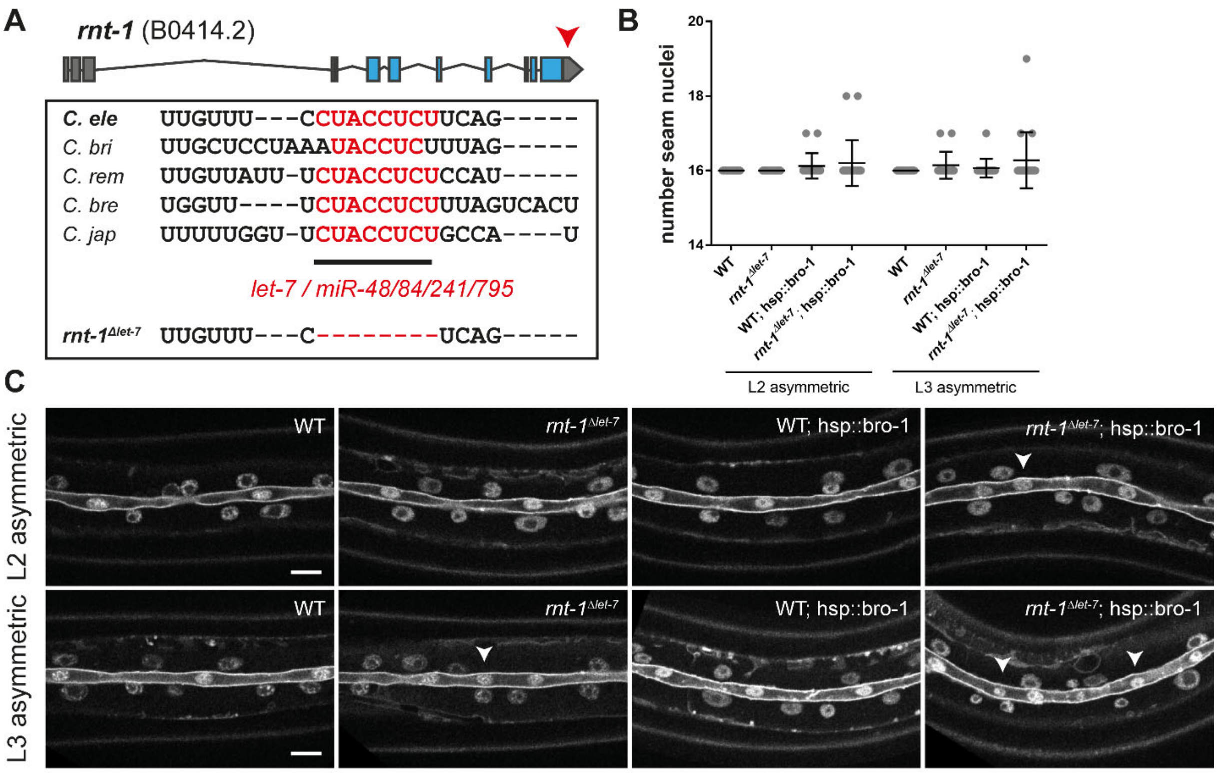

Figure S4. Removal of the *let-7s* miRNA target site in the endogenous RNT-1 3'UTR. (A) Illustration of the endogenous *rnt-1* 3' untranslated region (3'UTR; red arrowhead) with the *let-7* recognition sites marked in red. These sites are conserved between different nematodes (*C. briggsae, C. remanei, C. brenneri* and *C. japonica*). The red nucleotides were deleted from the endogenous locus using CRISPR/Cas9 recombineering. (B) Quantification of the number of seam nuclei in late L4 lateral seam syncytia of control, *rnt-1*$^{\Delta let\text{-}7}$, *hsp::bro-1* and *rnt-1*$^{\Delta let\text{-}7}$ *hsp::bro-1* animals subjected to heat shock prior to the L2 and L3 asymmetric divisions. (C) Spinning disk images of late L4 lateral seam syncytia of control, *rnt-1*$^{\Delta let\text{-}7}$, *hsp::bro-1* and *rnt-1*$^{\Delta let\text{-}7}$ *hsp::bro-1* animals subjected to heat shock prior to the L2 (left panel) and L3 (right panel) asymmetric divisions. Arrowheads indicate ectopic seam nuclei in the lateral syncytium. Images were processed using ImageJ software. Scale bars represent 10 µm. Error-bars indicate mean ± SD.

FIGURE S5

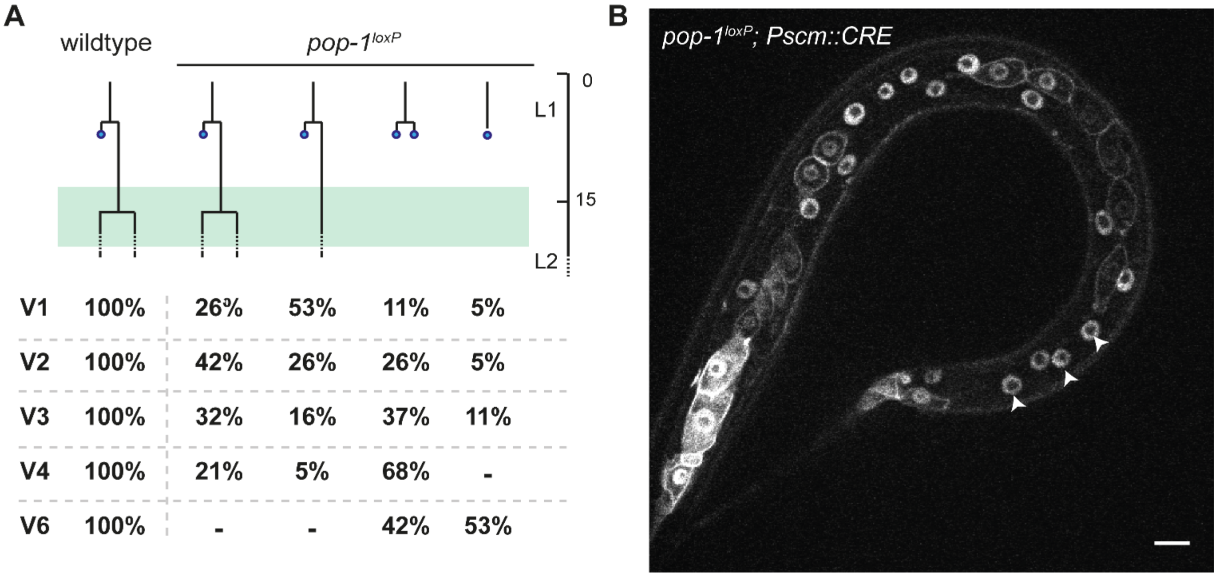

Figure S5. Conditional knock-out of *pop-1* reveals a dual role as transcriptional repressor and activator in seam cells. (A) Lineage analysis of *pop-1*^{loxP} early L2 animals undergoing the symmetric seam cell division. Y-axis represents developmental timing (hours; L2 starts at 15 hr). The four different lineages observed in *pop-1*^{loxP} animals are plotted against developmental time. The frequency of occurrence of each lineage was quantified for V1-V4 and V6 seam cells. Together, these quantifications reveal extensive premature differentiation of seam cells during L1 stage (B) Spinning disk confocal microscopy image of an L2 stage *pop-1*^{loxP}, P*scm::CRE* animals. Seam markers are mCherry::PH, mCherry::H2B. Arrowheads points to ectopic differentiation. Scale bar represents 10 µm.

Development • Supplementary information

FIGURE S6

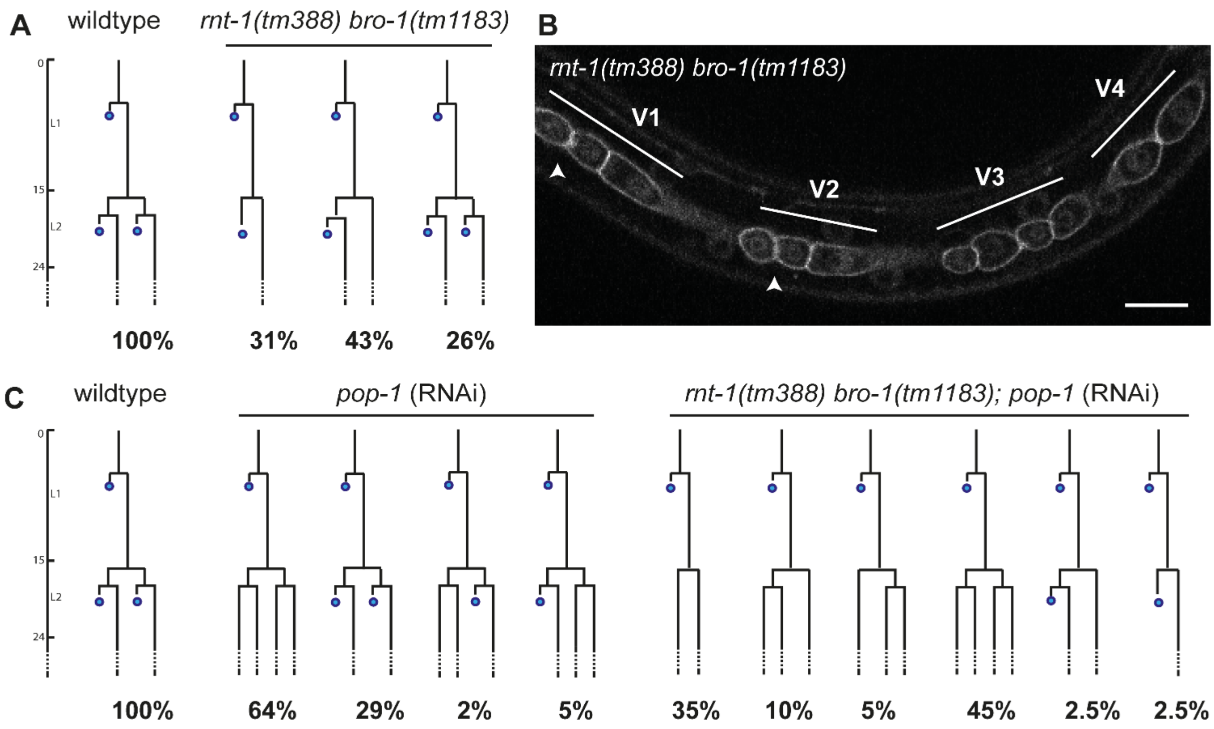

Figure S6. Overview of lineage analysis of wild-type and mutant strains. (A) Lineage analysis for wild-type animals and *rnt-1(tm388) bro-1(tm1183)* mutants performed at 20 °C. (B) Spinning disk confocal image of a *rnt-1(tm388) bro-1(tm1183)* animal. The seam markers used are GFP::PH and GFP::H2B. Arrowheads point to anterior daughter cells having undergone the L2 asymmetric division. Scale bar represents 10 µm. (C) Lineage analysis for wild-type animals, *pop-1(RNAi)* animals, and *rnt-1(tm388) bro-1(tm1183)* mutants exposed to *pop-1* RNAi by feeding.

Development • Supplementary information

FIGURE S7

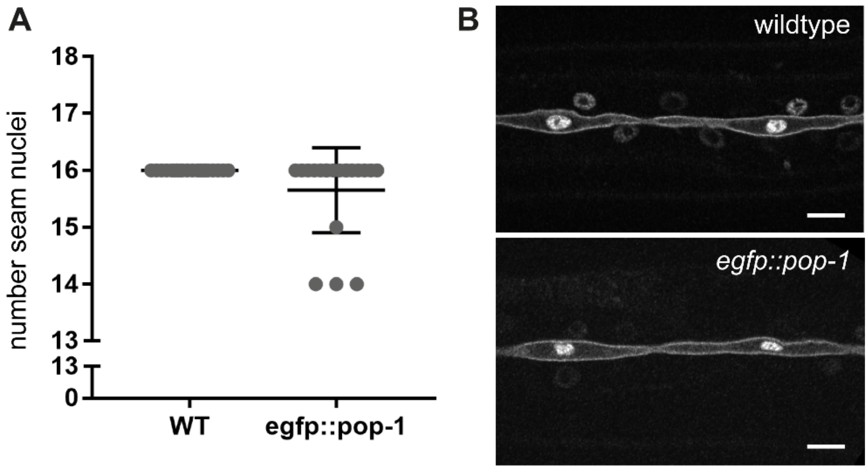

Figure S7. Characterization of seam epithelia in endogenous *egfp::pop-1* strain.
Quantification of seam cell nuclei in L4 seam syncytia in wild-type and knock-in animals (A). Representative spinning disk images of L4 stage seam syncytia of wild-type (top) and *egfp::pop-1* (bottom) animals (B). Images were processed with ImageJ software. Scale bars represent 10 µm. Error-bars represent mean ± SD.

FIGURE S8

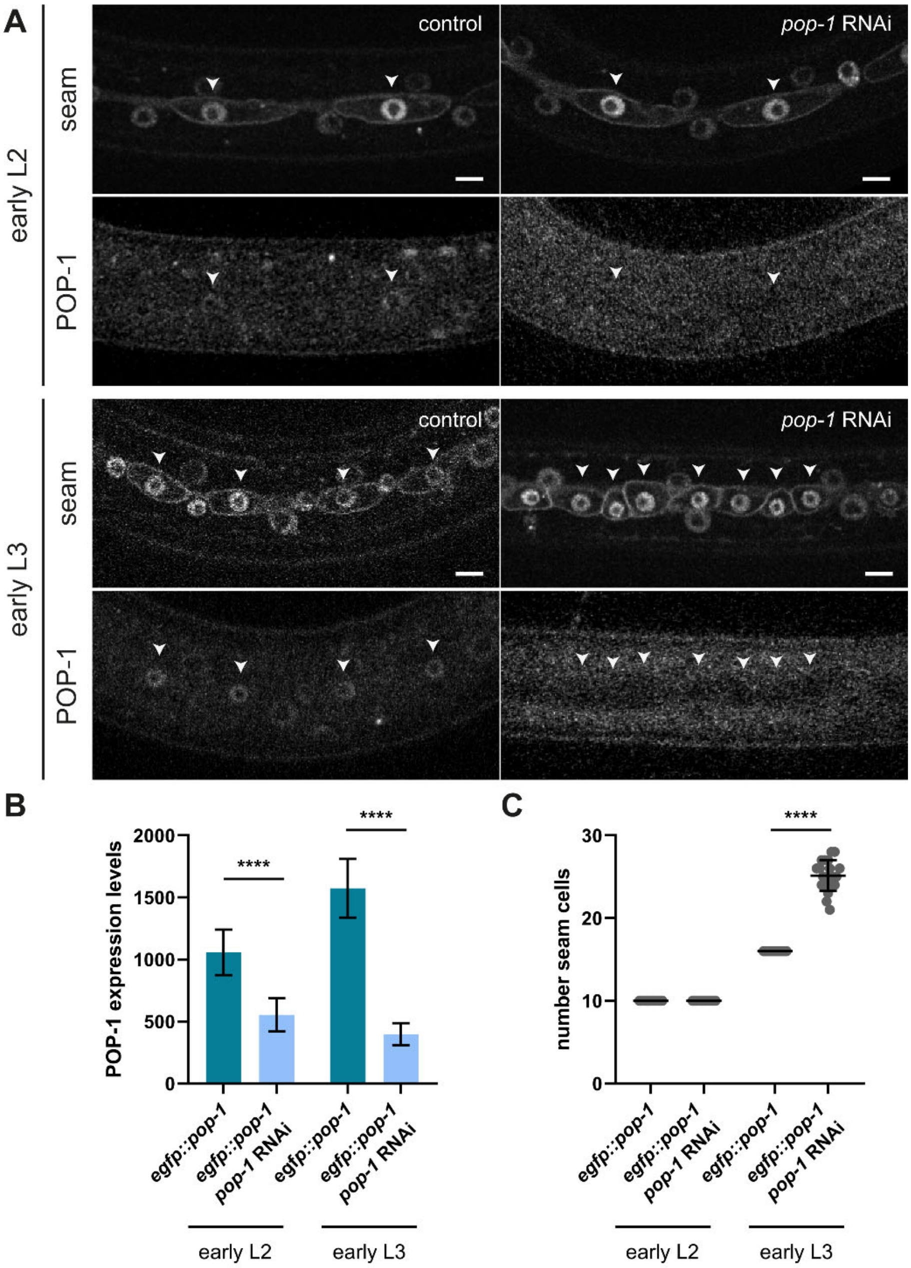

Figure S8. Endogenous *egfp::pop-1* animals treated with *pop-1 RNAi*. (A) Spinning disk images of early L2 stage (top) and early L3 stage (bottom) seam cells of control (left) and *pop-1* RNAi treated (bottom) *egfp::pop-1* animals. Arrowheads point to seam nuclei. (B) Quantification of the absolute nuclear EGFP::POP-1 levels in control and *pop-1* RNAi treated early L2 and early L3 animals. N $\geq$68. Levels are corrected for background outside of the animal in this experiment due to otherwise negative values in the RNAi treated cells. (C) Quantification of the number of seam cells in the same animals that were imaged in figures A and B. Images were processed with ImageJ software. Scale bars represent 10 µm. Error-bars represent mean ± SD.

FIGURE S9

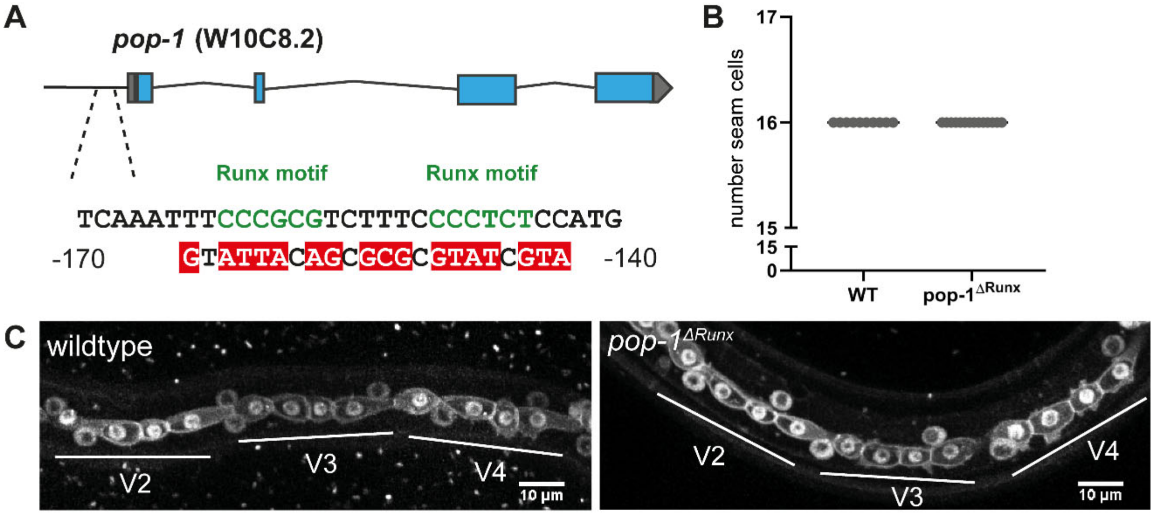

Figure S9. Mutation of candidate *rnt-1* binding sites in the endogenous *pop-1* promoter region is not sufficient to alter seam cell fate. Schematic representation of the pop-1 promoter region with the Runx binding motifs. Mutated residues are depicted in red (A). Quantification of seam cell numbers in late L2 animals of *pop1*$^{\Delta Runx}$ animals (B). Representative spinning disk images of *pop1*$^{\Delta Runx}$ late L2 stage seam cells (C). Images were processed using ImageJ software. Scale bars represent 10 µm. Error-bars represent mean ± SD.

Development • Supplementary information

FIGURE S10

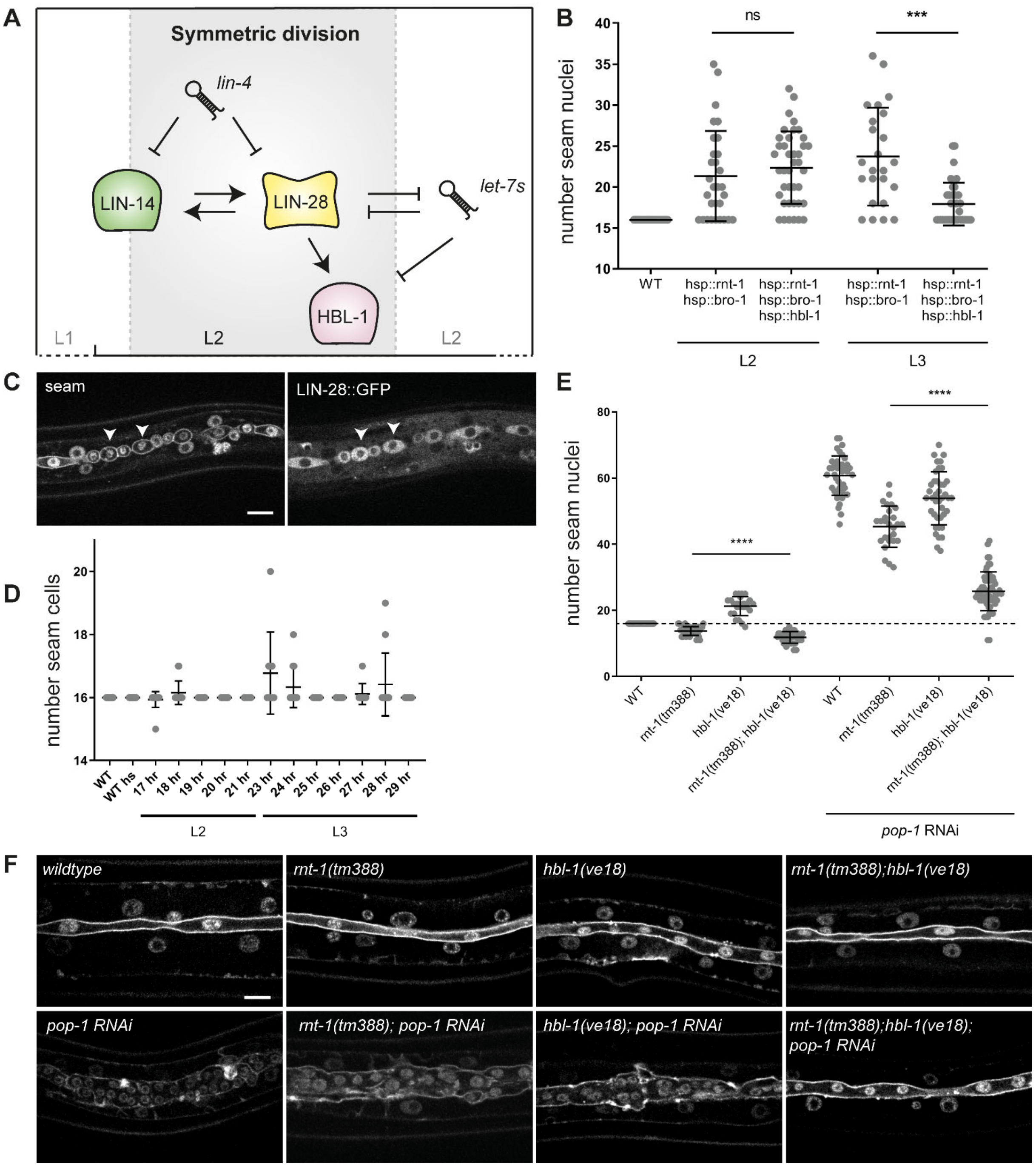

Figure S10. Manipulation of heterochronic *lin-28* and *hbl-1* gene expression during L2 and L3 asymmetric divisions. (A) Schematic cartoon of the L2 "symmetry window" as determined by expression of *lin-14*, *lin-28* and *hbl-1* genes. (B) Quantification of the number of seam cell nuclei in late L4 lateral seam syncytia, in animals subjected to heat-shock induction of *rnt-1 bro-1* and *rnt-1 bro-1 hbl-1* prior to the L2 asymmetric or L3 asymmetric division. (C) Spinning disk confocal microscopy image of an L3 larva subjected to heat-shock

Development • Supplementary information

induction of *lin-28::gfp*. Arrowheads point to posterior daughter cells of the L3 asymmetric seam cell division, displaying high cytoplasmic levels of LIN-28::GFP protein. (D) Quantification of seam cell numbers after heat-shock induction of *lin-28::gfp* in L2 (induction 17 hr-22 hr) or L3 larvae (induction 23-29 hr). Seam cells were counted at the end of L2 (t22 hr) or L3 (t30 hr). (E) Quantification of seam cell nuclei numbers in late L4 lateral seam syncytia of control, *rnt-1(tm388), hbl-1(ve18)* and *rnt-1(tm388);hbl-1(ve18)* animals with and without *pop-1(RNAi)*. (F) Spinning disk confocal microscopy images of late L4 lateral seam syncytia of control, *rnt-1(tm388), hbl-1(ve18)* and *rnt-1(tm388);hbl-1(ve18)* animals with and without *pop-1(RNAi)*. Images were processed using ImageJ software. Scale bars represent 10 µm. Error-bars indicate mean ± SD.

Table S1. Strains used in this study

Strain	Genotype
SV1009	*heIs63[*Pwrt-2*::gfp::ph;* Pwrt-2*::gfp::h2b;* Plin-48*::mcherry] V*
SV1984	*heIs218[*Pwrt-2*::mcherry::ph,*Pwrt-2*::mcherry::h2b,* Plin-48*::gfp] IV*
SV1986	*heIs218[*Pwrt-2*::mcherry::ph,* Pwrt-2*::mcherry::h2b,* Plin-48*::gfp] IV; qIs74[*Psys-1*::pop-1::gfp + unc-119(+)] him-5(e1490) V*
JK3437	*qIs74[*Psys-1*::pop-1::gfp + unc-119(+)] him-5(e1490) V;*
HS1486	*unc-76(e911) V; osIs13[*Papr-1*::apr-1::venus; unc-76 (+)]*
SV1615	*heIs188 [*Pwrt-2*::mcherry::ph;* Pwrt-2*::mcherry::h2b;* Plin-48*::gfp;* Phsp16.48*::cki-1::gfp]*
SV1694	*unc-119 (ed3) III ; heSi193 [*Pmcm-4*::CDK sensor::gfp::tbb-2 UTR + Cbr-unc119(+)] II ; dpy-20 (e1362) ; [*Pwrt-2*::mcherry::ph* Pwrt-2*::mcherry::h2b dpy-20(+)]*
SV2112	*pop-1(he334[pop-1^{loxP}]) I**
SV2113	*pop-1(he334[pop-1^{loxP}]) I; heIs218[*Pwrt-2*::mcherry::ph,* Pwrt-2*::mcherry::h2b,* Plin-48*::gfp] IV; heSi175(*Pscm*::cre) X*
SV2114	*pop-1(he335[egfp::loxP::pop-1]) I*
SV2115	*pop-1(he335[egfp::loxP::pop-1]) I; heIs218[*Pwrt-2*::mcherry::ph,* Pwrt-2*::mcherry::h2b,* Plin-48*::gfp] IV*
SV2000	*heIs63[*Pwrt-2*::gfp::ph;* Pwrt-2*::gfp::h2b;* Plin-48*::mcherry] V ; heEx609[*Phsp16.2*::rnt-1,* Phsp16.2*::bro-1,* Pmyo-2*::tdTomato]*
SV2002	*rnt-1(he305[rnt-1::egfp::3xflag::loxP]) I*
SV2003	*rnt-1(he305[rnt-1::egfp::3xflag::loxP]) I; heIs218[*Pwrt-2*::mcherry::ph;* Pwrt-2*::mcherry::h2b;* Plin-48*::gfp] IV*
YK138	*rnt-1(tm388) bro-1(tm1183) I*
SV2126	*rnt-1(tm388) bro-1(tm1183) I; heIs63[*Pwrt-2*::gfp::ph;* Pwrt-2*::gfp::h2b;* Plin-48*::mcherry] V*
AW811	*rnt-1(tm388) I; heIs63[*Pwrt-2*::gfp::ph;* Pwrt-2*::gfp::h2b;* Plin-48*::mcherry] V*
SV2148	*heSi193[*Pmcm-4*::cdk-sensor] II; heIs218[*Pwrt-2*::mcherry::ph,* Pwrt-2*::mcherry::h2b,* Plin-48*::gfp] IV; heEx609[*Phsp16.2*::rnt-1,* Phsp16.2*::bro-1,* Pmyo-2*::tdTomato]*
SV2150	*pop-1(he335[egfp::loxP::pop-1]) I; heIs218[*Pwrt-2*::mcherry::ph,* Pwrt-2*::mcherry::h2b,* Plin-48*::gfp] IV; heEx609[*Phsp16.2*::rnt-1,* Phsp16.2*::bro-1,* Pmyo-2*::tdTomato]*
SV2230	*pop-1(he335[egfp::loxP::pop-1] rnt-1(tm388) bro-1(tm1183)) I; heIs218[*Pwrt-2*::mcherry::ph,* Pwrt-2*::mcherry::h2b,* Plin-48*::gfp] IV*
SV2231	*pop-1(he373[pop-1$^{\Delta Runx}$] I**; heIs63[*Pwrt-2*::gfp::ph;* Pwrt-2*::gfp::h2b;* Plin-48*::mcherry] V*
YK152	*bro-1(tm1183) I, Is[*Pbro-1*::bro-1::gfp]*

*loxP sites are present upstream of the *pop-1* ATG and in intron 5

**promoter sites were altered between 143 and 164bp upstream of the *pop-1* ATG start codon (See Fig. S5)

Development • Supplementary information

Table S2. Overview of oligonucleotides used in this study

Oligo	Sequence
pop-1^{loxP}	
pop-1 N-terminus gRNA 1	ctcatcgccgagctcttcgtcgg
pop-1 N-terminus gRNA 2	tttttgtgtatttttatatctgg
pop-1 C-terminus gRNA 1	taaatgtctactgtagcggaagg
pop-1 C-terminus gRNA 2	cgctacagtagacatttatgggg
pop-1 N-terminus repair ssDNA oligo	cgttaaaaaatgctctaaatttcaagatataaaaatacacataacttcgtatagcatacattatacgaagttataaaaatgatggcagacgaagagctcggcgatgaggtgaag
pop-1 C-terminus repair ssDNA oligo	ttcatttttctacatcacatgaataacaccataaatgtctataacttcgtatagcatacattatacgaagttatactgtagcggaaagaaaaattaacagcgctacggtagtca
pha-1 repair ssDNA oligo	caaaatacgaatcgaagactcaaaaagagtatgctgtatgattacagatgttcatcaagttattcataaatcattgatag
egfp::pop-1	
pop-1 exon 1 gRNA 1	gctcatcgccgagctcttcgtcgg
pop-1 LHA FW	ggctgctcttcgtggttggtagaagtctaaacctccacttt
pop-1 LHA RV	gggtgctcttcgcatttttgtgtatttttatatctgg
pop-1 RHA FW	agagctcggcgatgaggtgaaagtgttccgtcgggatgagg
pop-1 RHA RV	gggtgctcttcgtacgaactccgcccataaaaccgt
pop-1$^{\Delta Runx}$	
pop-1 N-terminus gRNA 1	catggagaggggaaagacgcggg
pop-1 N-terminus gRNA 2	cggaagttaggccatggagaggg
pop-1 promoter repair oligo	aacctccactttctccccaaaatcctatcgaattcaaatgtattacagcgcgcgtatcgtaatgccctaacttccgcggacctagtccccctttttcttgttttaaatggttcc
rnt-1::egfp	
rnt-1 C-terminus gRNA 1	atagttcttctccgactatttgg
rnt-1 C-terminus gRNA 2	gttcttctccgactatttggagg
rnt-1 LHA FW	cagatgccaatgacaatgattccacc
rnt-1 LHA RV	aaaaggtctccatatcgtaggtgatgagctattcgatgaagt
rnt-1 RHA FW	tcttaaaaatattcattattttaccacaacacacc
rnt-1 RHA RV	tctaatcatccatctcccaactc

Development • Supplementary information

**Heat shock expression**	
FW *hsp16.48*	ctggacggaaatagtggtaaag
RV *hsp16.48*	tcttgaagtttagagaatgaacag
FW *unc-54 UTR*	catctcgcgcccgtgcc
RV *unc-54 UTR*	aaacagttatgtttggtatattggg
FW *cki-1*	atgtcttctgctcgtcgttg
RV *cki-1*	gtatggagagcatgaagatcg
FW *egfp*	atgtccaagggagaggagc
RV *egfp*	ttacttgtagagctcgtccattcc

Development • Supplementary information
